# Study of Internal Stress in Conductive and Dielectric Thick Films

**DOI:** 10.3390/ma15238686

**Published:** 2022-12-06

**Authors:** Jiri Hlina, Jan Reboun, Martin Janda, Ales Hamacek

**Affiliations:** Department of Materials and Technology, Faculty of Electrical Engineering, University of West Bohemia, Univerzitni 8, 301 00 Pilsen, Czech Republic

**Keywords:** thick film, silver, dielectric, internal stress, ceramics, cantilever method

## Abstract

This paper is focused on the study of internal stress in thick films used in hybrid microelectronics. Internal stress in thick films arises after firing and during cooling due to the differing coefficients of thermal expansion in fired film and ceramic substrates. Different thermal expansions cause deflection of the substrate and in extreme cases, the deflection can lead to damage of the substrate. Two silver pastes and two dielectric pastes, as well as their combinations, were used for the experiments, and the internal stress in the thick films was investigated using the cantilever method. Further experiments were also focused on internal stress changes during the experiment and on the influence of heat treatment (annealing) on internal stress. The results were correlated with the morphology of the fired thick films. The internal stress in the thick films was in the range of 8 to 21 MPa for metallic films and in the range from 12 to 16 MPa for dielectric films. It was verified that the cantilever method can be successfully used for the evaluation of internal stress in thick films. It was also found that the values of deflection and internal stress are not stable after firing, and they can change over time, mainly for metallic thick films.

## 1. Introduction

Thick film technology is well known and has been used for a very long time in various electronic fields for the production of hybrid circuits or passive components [1]. This technology is used for the creation of conductive, resistive, or dielectric films, which are printed in the form of paste on a ceramic substrate and usually fired in belt furnaces [2]. Thick film pastes contain several components that ensure the properties of the final film, including good adhesion on the substrate, as well as the good printing properties of the paste [3]. Thick films represent simple mass-scalable technology, compared to complex thin film technology, which is based on physical vapor deposition (PVD) or chemical vapor deposition (CVD) methods [4].

Films of different materials deposited on substrates create tensile or compressive internal stress [5,6]. This stress causes the deflection of the substrate, creating problems with the printing of additional layers on an uneven substrate, assembling electronic components, and the integration of the substrate into the final device. In extreme cases, internal stress may lead to the formation of cracks in the film, film delamination, or even substrate damage. The internal stress in the films is related to the process of their formation, to the physical and chemical reactions during the formation of the film, and in particular, to the different thermal expansion coefficients of the materials used (film and substrate) [7,8,9,10]. Most films are formed at a temperature higher than room temperature, and as the temperature decreases, additional internal stress is created in the film. Internal stress also affects the physical properties of the films [11,12,13,14]. The magnitude of the internal stress can also change over time, and therefore, the properties of the film can change as well [15]. This may be caused by relaxation effects, the creep of material in a solid state in some components of the film, the diffusion of defects in the crystal lattice, grain boundary effects, etc. [16,17,18,19,20].

Thick films are sintered (fired) at high temperatures (usually 850 °C), at which time, the films are compacted [21]. During the cooling of the films to room temperature, their shrinkage occurs [22]. This shrinkage is limited by the ceramic substrate on which the films are deposited. Because shrinkage is not allowed in the plane of the film, tensile stress arises in the film, which causes the substrate to deflect, which can cause delamination or crack formation [23]. In multilayer structures, internal stress can arise not only due to the differences in the thermal expansion of the films and substrates, but also due to different thermal expansion of the subsequently individual deposited films during high-temperature heat treatment.

Deflection of the substrate during cooling is much more enhanced in the case of thick film power electronic substrates, where the higher thickness of films is required for high current capability and good heat dissipation from the loaded electronic components [24]. Special silver or copper thick film pastes are used for the realization of these substrates. Power electronic substrates with copper films are called thick printed copper (TPC) substrates [25]. TPC technology enables the realization of copper films with thicknesses up to 300 µm, and this thickness can cause significant deflection of the ceramic substrate [26]. Therefore, the proper design of copper patterns and similar pattern areas on both sides of the substrate is necessary for the limitation of deflection.

In the literature, only methods for the measurement of internal stress in thin films in the field of hybrid integrated circuits and semiconductor chips are described. The main reason for the measurement of internal stress in thin films is the possibility of significant defect formation due to high internal stress in the range of GPa [27,28,29,30] and the fact that the inner structure of thin films is much more homogeneous compared to that of thick films, which contain more components and pores, and their surface is not smooth [4,31]. Internal stress in thin films can be measured by the following methods: laser scanning, multi-beam optical stress sensor, grid reflection, coherent gradient sensor (CGS), X-ray diffraction, and cantilever [32,33,34,35,36,37,38,39]. The literature describes the maximum radius of curvature that can be detected by some of these methods—10^4^ m for the laser scanning method, 2.5 × 10^4^ m for the multi-beam optical stress sensor method, and 1.25 × 10^3^ m for the CGS method [32,40,41]. Except for the cantilever method, all of the other methods require the use of special measurement devices. Methods for the measurement of internal stress dedicated directly to thick films have not yet been described in the literature. Therefore, this paper is focused on the issue of internal stress in thick films, along with its measurement.

## 2. Materials and Methods

The methodology proposed for the measurement of internal stress in thick films is based on the ASTM D6991 standard test method for the measurement of internal stresses in organic coatings using the cantilever method [42]. The cantilever method can be easily implemented in laboratory conditions, as well as in an industrial environment, without the need for special equipment. This method is based on the measurement of the deflection of the substrate with the printed film, which is clamped in the special fixture. Internal stress S in the thick film is calculated according to the following formula:

S = (hE_s_t^3^)/(3L^2^c(t + c)(1 − γ_s_))         (MPa), (1)

where h is the deflection of the cantilever (in mm), E_S_ is the modulus of elasticity of the substrate (for alumina 300 GPa [43]), t is the thickness of cantilever substrate (in mm), L is the length of the substrate between the edge point at which it is clamped and point of which deflection is measured (in mm), c is the thickness of the film (in mm), and γ_S_ is Poisson’s ratio of the cantilever substrate (for alumina 0.22 [43]).

**Figure 1:**
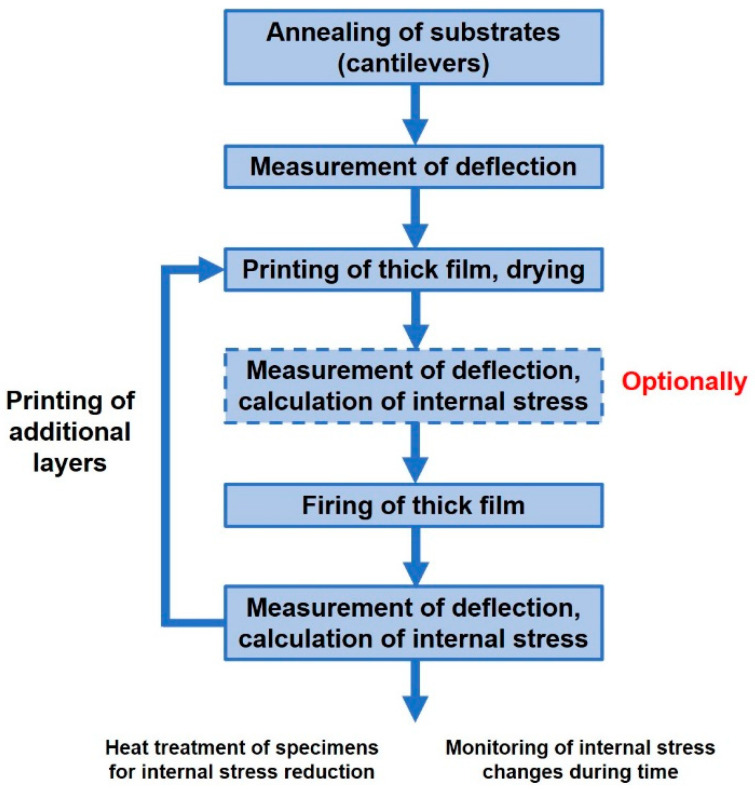
Procedure for the measurement of internal stress in thick films.

The individual steps for specimen preparation and internal stress measurement are summarized in Figure 1. The first step is the annealing of substrates (cantilevers) at the temperature specified for firing the thick film and the measurement of deflection in the fixture. This deflection value represents zero value for the next measurements, after printing the films. Then, following the printing (screen printing or stencil printing) of the film on the cantilever, its drying and optional measurement of deflection after drying and calculation of internal stress is performed, according to formula 1. The calculation of internal stress after drying was used only for the verification of the drying process, and is not important for the final internal stress measurement. The printed film is then fired, followed by the measurement of the final cantilever deflection and the calculation of internal stress. The next steps can include the printing of additional layers, the heat treatment (annealing) of specimens for internal stress reduction, or the monitoring of internal stress changes over time.

**Figure 2:**
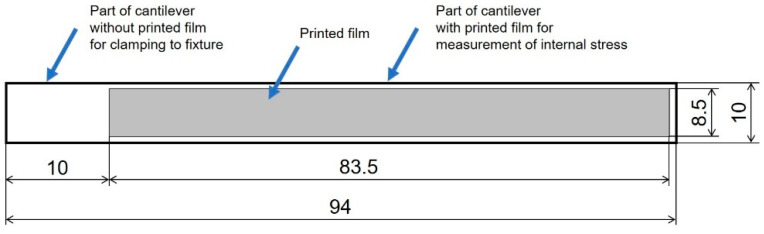
Dimensions of the substrate (cantilever) and the printed pattern.

Alumina substrates (96% Al_2_O_3_) with a thickness of 0.318 mm were used for the preparation of test specimens as cantilevers. The dimensions of the alumina substrate, as well as the dimensions of the printed film pattern, are shown in Figure 2. The printed pattern does not cover the whole substrate, and the uncovered area of substrate on the left is intended for clamping the fixture. 

**Figure 3:**
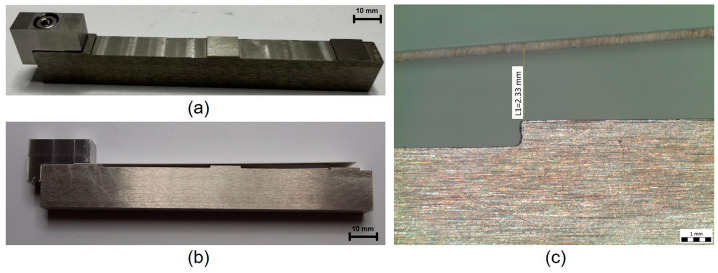
(**a**) Fixture for clamping of the test specimen; (**b**) fixture with clamped substrate (cantilever); (**c**) measurement of clamped substrate deflection by stereomicroscope.

A special fixture for the measurement of internal stress in thick films using the cantilever method (Figure 3) was designed and manufactured. This fixture was manufactured from stainless steel and consists of two parts—a fixture body, with a marked measuring point of deflection at a distance of 80 mm from the edge point of clamping, and a part for the clamping of the cantilever.

Four commercially available thick film pastes were chosen for the experiments regarding the measurement of internal stress in thick films—two widely-used conductive pastes, and two dielectric pastes. The conductive pastes were silver paste Heraeus C8717E (drying temperature 150 °C, firing temperature 850 °C) and silver-platinum paste Heraeus C1076SD (drying temperature 150 °C, firing temperature 850 °C). The dielectric pastes were glass overglaze paste DuPont QQ550 (drying temperature 150 °C, firing temperature 525 °C) and multilayer dielectric paste DuPont 5704 (drying temperature 150 °C, firing temperature 850 °C). These pastes are designed for firing in an oxidative atmosphere.

The above-mentioned thick film pastes were printed on alumina substrates (cantilevers) using stencil printing with an 80 µm stainless steel stencil. Eight groups of specimens were realized in total, with various combinations of printed films. These specimen groups are described in Table 1. This table also contains the number of specimens in each group. Specimens in groups 1–4 contained one printed layer of each paste. Specimens in groups 5 and 6 had two sequentially printed and fired layers of silver pastes C8717E and C1076D. These two specimen groups with high film thickness (~130 µm) represent the use of thick films for power applications. Specimens in groups 7 and 8 were created with a combination of conductive and dielectric pastes. The first layer was printed with C8717E or C1076SD paste and then dried and fired. The second layer was printed with glass overglaze paste QQ550. These samples represent a combination of conductive and dielectric films for real applications, where the silver film can be covered with glass overglaze.

**Figure 4:**
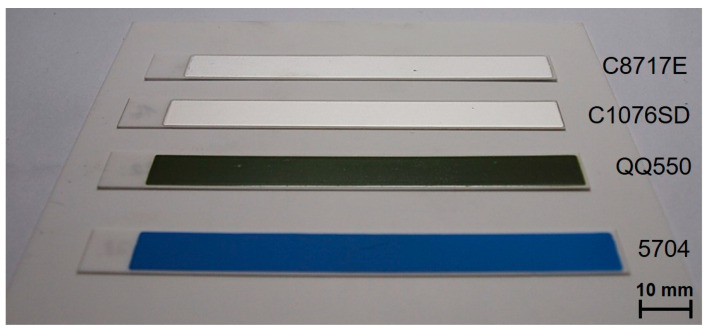
Fired specimens—groups 1–4.

The printed films were fired in a chamber furnace, and two firing profiles were used—a profile with a 850 °C peak temperature, with a 10 min dwell time at peak temperature for pastes C8717E, C1076SD, and 5704, and a profile with a 525 °C peak temperature, with a 2 min dwell time at peak temperature for paste QQ550. The fired specimens for groups 1–4 are shown in Figure 4. The average thickness of the printed films (Table 1) was calculated from five values measured on cross-sections by SEM microscope Phenom ProX. The realized specimens were clamped to the fixture and deflection of the cantilever was measured by stereomicroscope Olympus SZX10. The accuracy of the optical measurement is ±0.01 mm, which corresponds with the accuracy of ±0.3 MPa for the 100 µm film thickness. Higher accuracy can be achieved by a longer cantilever or by a microscope with higher resolution. The maximum curvature that can be measured using the described cantilever was calculated to be 0.32 × 10^3^ m. The accuracy of the method described in this study is sufficient for the internal stress measurement of thick films. The internal stress in thick films was calculated according to Formula (1).

Additional experiments were dedicated to the measurement of internal stress changes over time. Internal stress was measured after 1000, 2000, and 3000 h from firing (initial values). Heat treatment (annealing) of test specimens which could theoretically reduce the internal stress, was applied after these experiments. This annealing was carried out at the recommended firing temperature of each paste, with a two-hour dwell time.

## 3. Results and Discussion

**Figure 5:**
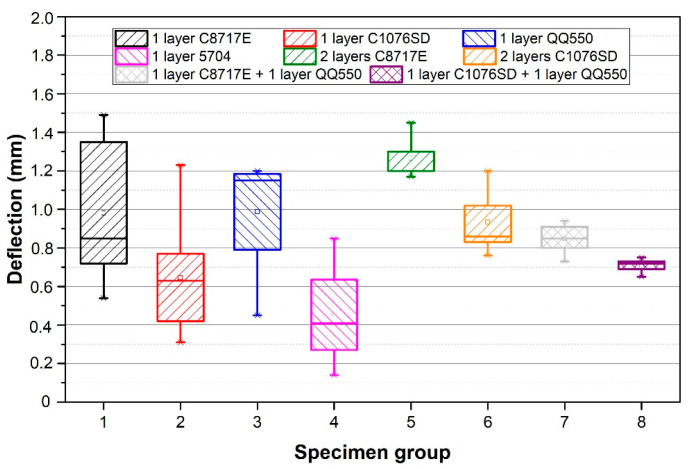
Results of deflection measurement for all specimen groups.

**Figure 6:**
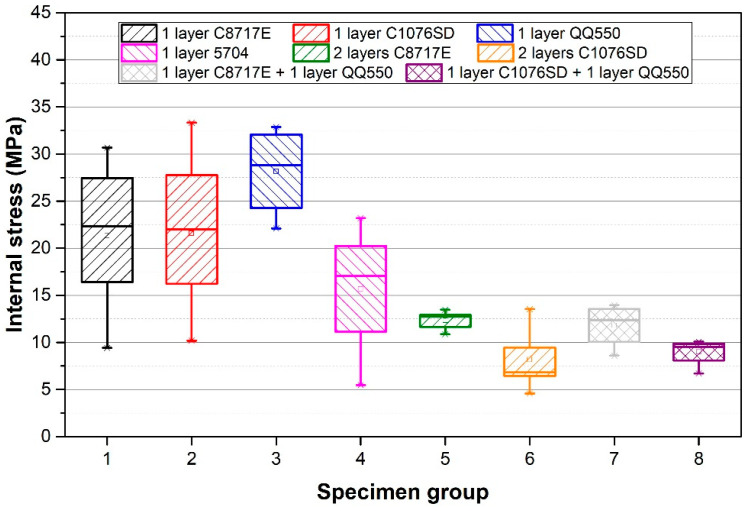
Calculated internal stress for all specimen groups.

Results of the deflection measurement of all specimen groups are described in Figure 5, and the results of the internal stress, calculated according to Formula 1, are shown in Figure 6. The calculated internal stress after firing, internal stress changes over time, and internal stress after two-hour annealing are described in Table 2.

The firing cycle of the printed films is divided into two parts—heating and cooling. Directly after the printing, the film is not compact, and during the heating, it is not yet connected to the ceramic substrate. Therefore, different coefficients of thermal expansion for the film and substrate are not reflected in this part of the firing cycle, and no internal stress is formed. The melting of glass binder particles occurs at a temperature above 500 °C, and silver particle sintering occurs rapidly at temperatures above 800 °C. During cooling, the film becomes compact, and the connection between the film and the ceramic substrate is created and fixed. The compact layer cannot yet absorb temperature stress, and it shrinks more during cooling due to its higher CTE (8.0 × 10^−6^ K^−1^ for Al_2_O_3_, 22.4 × 10^−6^ K^−1^ for silver, and 4–9.1 × 10^−6^ K^−1^ for glass [22,43,44,45,46]). Therefore, the substrate with the film deflects upwards (towards the printed film), and tensile stress is formed.

**Figure 7:**
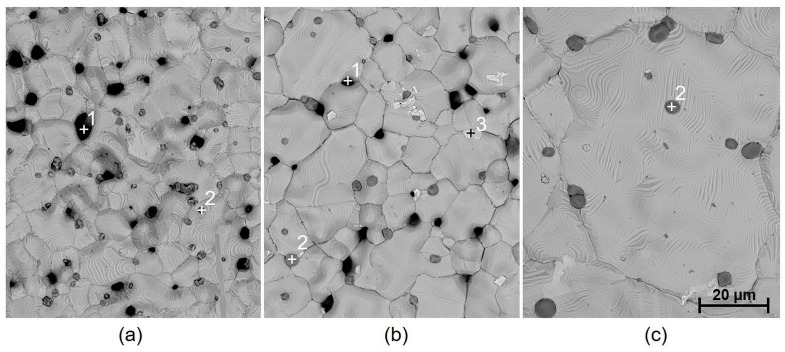
SEM image of the surface of fired paste C8717E: (**a**) 1 layer and 1 firing (specimen group 1), (**b**) 2 layers and 2 firings (specimen group 5), and (**c**) 2 layers and 2 firings, flowed by a two-hour annealing (850 °C). Spot 1, pores; spot 2, Cu_2_O; spot 3, Bi_2_O_3_, SiO2, and Al_2_O_3_.

**Figure 8:**
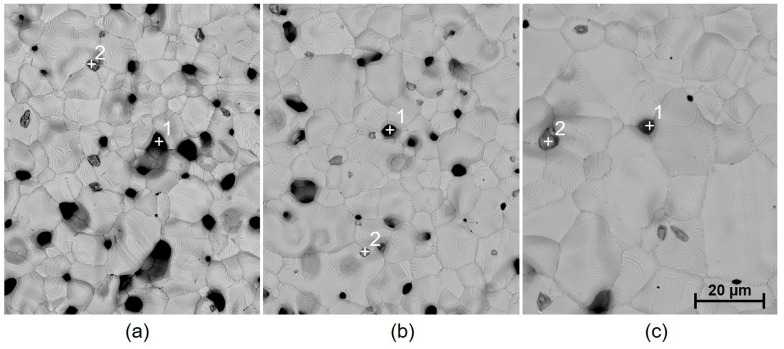
SEM image of the surface of fired paste C1076SD: (**a**) 1 layer and 1 firing (specimen group 2), (**b**) 2 layers and 2 firings (specimen group 6), and (**c**) 2 layers and 2 firings, followed by a two-hour annealing (850 °C). Spot 1, pores; spot 2, Cu_2_O.

The deflection of specimens with one silver layer (specimen groups 1 and 2) is higher in the case of paste C8717E, while internal stress is similar for both pastes C8717E and C1076SD. The slightly higher deflection of the specimens with paste C8717E compared to specimens with paste C1076SD can be caused by different adhesion mechanisms. The adhesion of paste C8717E is based on glass frit, which penetrates the ceramic substrate during the firing cycle, while the adhesion of paste C1076SD is based on metal oxides. In the case of specimens with two silver layers (specimen groups 5 and 6), the deflection is higher compared to that of specimens with one silver layer, owing to the higher thickness of silver films. On the contrary, the calculated internal stress is lower due to the higher thickness, according to Formula 1. Higher deflection is also caused by the more compact structure of silver films created by printing and firing the second silver layer, with fewer pores (comparison of SEM images of fired film with one and two silver layers in Figure 7 and Figure 8). In the case of paste C8717E, the deflection is enhanced by the more limited penetration of the glass phase into the substrate during the firing of the second layer [47]. The deflection of the two-layer specimens is higher for paste C8717E than for paste C1076SD, because the C8717E paste has larger grains, thus producing a more compact film with greater shrinkage during cooling after firing. Silver films contain not only silver grains, but also additives for promoting adhesion and sintering, as well as pores (black spots in SEM images). Adhesion and sintering promoters are based on Bi_2_O_3_, SiO_2_, and Al_2_O_3_, represented by white spots in Figure 7, and Cu_2_O, represented by dark grey spots in Figure 7 and Figure 8.

Differences in deflection and internal stress were observed in the case of the dielectric pastes (specimen groups 3 and 4). Specimen with dielectric paste 5704 exhibit the lowest deflection and internal stress of all the specimens with one printed layer. The thermal expansion of this paste is better matched to alumina ceramics. EDS analysis of dielectric pastes proved that the composition of paste 5704 is based mainly on aluminum and silicon oxides (36.6 wt. % Al_2_O_3_, 29.1 wt. % SiO_2_, 11.9 wt. % PbO, 10.7 wt. % CaO, 7.8 wt. % ZrO_2_, 2.0 wt. % MgO, and 1.9 wt. % Na_2_O), while the composition of paste QQ550 is based on lead glass (82.8 wt. % PbO, 15.6 wt. % SiO_2_, and 1.6 wt. % Al_2_O_3_).

**Figure 9:**
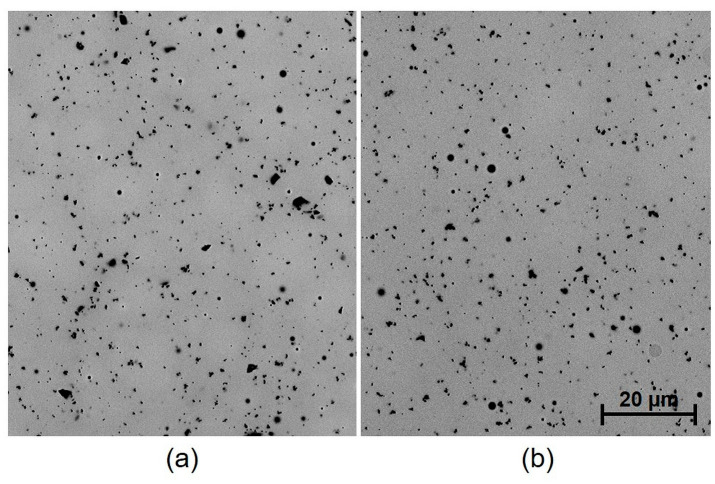
SEM image of the surface of fired paste QQ550: (**a**) 1 layer and 1 firing (specimen group 3); (**b**) 1 layer and 1 firing, followed by a two-hour annealing (525 °C).

**Figure 10:**
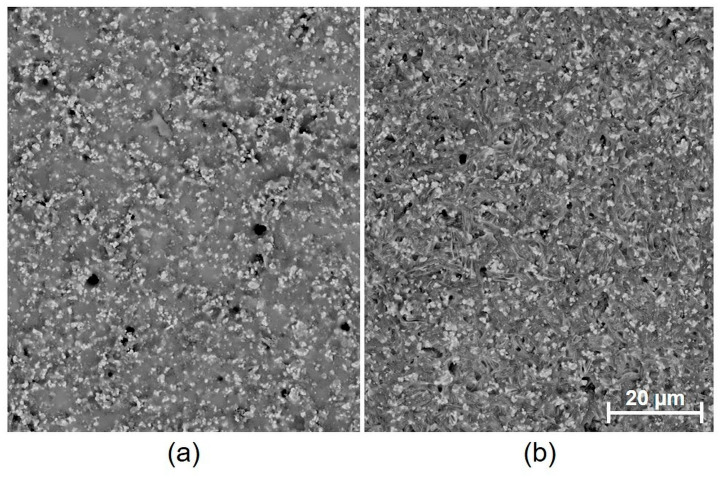
SEM image of the surface of fired paste 5704: (**a**) 1 layer and 1 firing (specimen group 4); (**b**) 1 layer and 1 firing, followed by a two-hour annealing (850 °C).

It was found that not only the paste material and the number of firings affect the internal stress value, but that the dwell time at peak temperature also has a significant influence. The presumption that internal stress release could occur during heat treatment (annealing) has not been proven (except for regarding the dielectric paste 5704). On the contrary, the annealing resulted in a significant increase in both deflection and internal stress. This effect was observed only for the metallic films. From the microscopic images, it is obvious that during annealing (2 h dwell time at a firing temperature 850 °C), there is a significant growth of silver grains from an initial size of 5–10 µm to a final size of 20–80 µm, depending on the paste type (Figure 7 and Figure 8). This causes the film to become even more compact, containing fewer pores, which results in higher deflection after cooling. In the case of Ag-Pt paste C1076SD, the deflection is lower, which corresponds to the lower grain size after annealing. Different effects were observed for dielectric materials. For dielectric paste QQ550, no changes were observed in the film morphology (Figure 9), or in the deflection and internal stress values after annealing at the maximum process temperature (2 h dwell time at a firing temperature of 525 °C). This paste is stable up to a temperature of 525 °C and does not crystallize. For dielectric paste 5704, a crystallization effect was observed at an annealing temperature of 850 °C for 2 h (Figure 10), which has a positive effect on both deflection and internal stress values. The deflection is levelled, and the internal stress is reduced after annealing.

**Figure 11:**
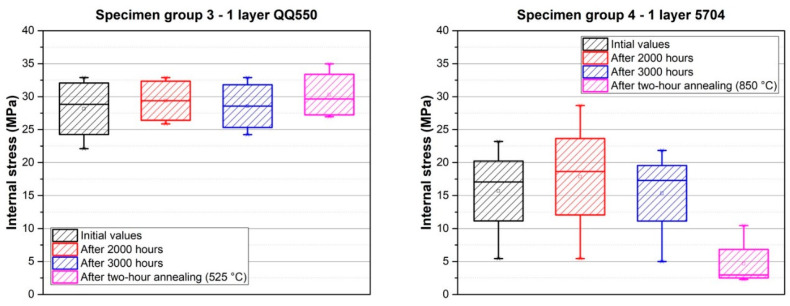
Internal stress changes over time for dielectric films—specimen group 3 (**left**), and specimen group 4 (**right**).

**Figure 12:**
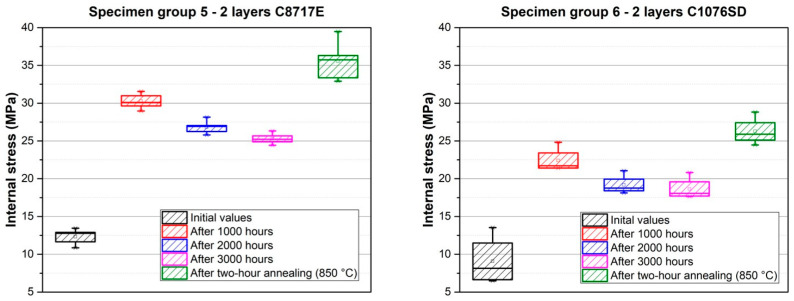
Internal stress changes over time for silver films—specimen group 5 (**left**), and specimen group 6 (**right**).

**Figure 13:**
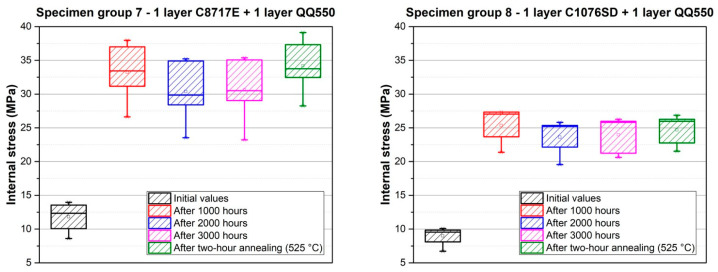
Internal stress changes over time for the combination of silver films and overglaze paste QQ550—specimen group 7 (**left**), and specimen group 8 (**right**).

Further experiments were focused on the measurement of internal stress changes over time at a temperature of 22 °C. This measurement was carried out for specimen groups 3–8. The results are described in Figure 11, Figure 12 and Figure 13 and also in Table 2. Internal stress was measured after firing, and these values represent the initial values in the following graphs. The internal stress was then measured 1000 (except for specimen groups 3 and 4), 2000, and 3000 h after firing. The values of internal stress after two-hour annealing, which was performed on the same specimens after the measurement of internal stress changes over time, are also shown in Figure 11, Figure 12 and Figure 13.

The results of internal stress changes over time for the dielectric films are shown in Figure 11. It is obvious that internal stress, in the case of overglaze paste QQ550 (specimen group 3), is stable over time, and its values are around 28.3 MPa. Moreover, the two-hour annealing at 525 °C did not cause any significant internal stress changes. These results corresponds with the results of the microscopic analysis, which showed no changes in film morphology after two-hour annealing (Figure 9). The internal stress, in the case of multilayer dielectric paste 5704, is also stable over time, and its values are in the range of 15–18 MPa. Different behavior has been observed after two-hour annealing (850 °C); there was a significant decrease in internal stress (2 MPa). This also corresponds with the results of the microscopic analysis, which showed a recrystallization effect after two-hour annealing (Figure 10).

The results of internal stress changes over time for silver films are described in Figure 12. Both films show a similar character regarding stress changes, with a significant increase in internal stress after 1000 h, compared to the initial values after firing (an increase of ~18 MPa from 12.3 MPa for paste C8717E, and an increase of ~14 MPa from 8.2 MPa for paste C1076SD). After this increase, a gradual stabilization of internal stress was observed. After 3000 h, internal stress values were stabilized at ~25.5 MPa for paste C8717E and ~18.6 MPa for paste C1076SD. Moreover, a further increase in internal stress occurred after the two-hour annealing at 850 °C. The increasing internal stress after two-hour annealing was caused by the significant growth in silver grains size, which has been proven by microscopic analysis (Figure 7 and Figure 8).

The result of internal stress changes over time for the combination of silver films and glass overglaze paste QQ550 are shown in Figure 13. A combination of silver films covered with glass overglaze is commonly used in real applications, in which glass overglaze protects the silver film against the environment. In the case of these samples, the same behavior is visible as that observed for individual silver films (specimen groups 5 and 6). There was also a significant increase in internal stress after 1000 h, compared to the initial values after firing (an increase of ~21.5 MPa from 11.8 MPa for a combination of pastes C81717E and QQ550 and an increase of ~16.5 MPa from 9.0 MPa for the combination of pastes C1076SD and QQ550). After this increase, a gradual stabilization of internal stress was also observed after 3000 h, as in the case of silver films at the value of ~30.6 MPa for the combination of C8717E and QQ550 and a value of ~24.7 MPa for the combination of C1076SD and QQ550. Two-hour annealing at 525 °C did not cause significant changes in internal stress compared to that of the individual silver films without overglaze. It is caused by the fact that these specimens were annealed at the lower temperature of 525 °C, which is the recommended temperature for the firing of overglaze paste QQ550.

## 4. Conclusions

It was verified that the cantilever method, which was originally used for internal stress measurement in organic coatings, can be also successfully used for the evaluation of internal stress in thick films. This method is suitable for use in laboratory conditions, as well as on an industrial scale for the fast, simple, and effective characterization of thick films by optical observation. The maximum radius of curvature that can be measured using the described cantilever method is slightly lower than that of more sophisticated methods requiring the use of special equipment and intended mainly for the measurement of internal stress in thin films. The accuracy of the cantilever method described in this study is sufficient for the internal stress measurement of thick films. The methodology for the measurement of internal stress in thick films using this method was designed and validated.

The values for calculated internal stress correspond well with the morphology changes in the thick films. If the grains grow in metallic films and the number of pores is reduced, the internal stress values visibly increase. On the contrary, when recrystallization occurs in the dielectric films, the internal stress and deflection values are reduced. It was observed that the heat treatment (annealing) of the metallic films at a maximum process temperature did not cause any reduction in internal stress, but rather, caused the opposite effect. This is caused by the significant growth of silver grains in annealed film (from an initial size of 5–10 µm to a final size of 20–80 µm, depending on the paste type). Annealing causes the densification of the films, which results in higher deflection and internal stress. It was also found that, after firing, the deflection and internal stress values are not stable and can change over time, mainly for the metallic thick films. This fact could cause serious reliability issues regarding the final devices. The issue of excessive substrate deflection and internal stress is becoming an important topic due to the increasing number of thick film substrate applications in power electronics, where conductivity patterns are up to 300 µm thick and are highly compact. Therefore, it is very important to measure the deflection and internal stress after firing, as well as to consider the evolution of both over time.

## Figures and Tables

**Table 1:** Description of test specimen groups.

Specimen Group	Paste		Total Film Thickness (µm)	Number of Specimens (-)
	First Layer	Second Layer		
1	Ag C8717E	-	66	15
2	Ag-Pt C1076SD	-	55	15
3	Dielectric QQ550	-	37	5
4	Dielectric 5704	-	42	5
5	Ag C8717E	Ag C8717E	133	5
6	Ag-Pt C1076SD	Ag-Pt C1076SD	129	5
7	Ag C8717E	Dielectric QQ550	101	5
8	Ag-Pt C1076SD	Dielectric QQ550	120	5

**Table 2:** Results of internal stress measurement.

Specimen Group	Internal Stress after Firing (Mpa)	Internal Stress Changes Over Time (Mpa)			Internal Stress after Two-Hour Annealing (Mpa)
		After 1000 h	After 2000 h	After 3000 h	
1	21.30	-	-	-	-
2	19.81	-	-	-	-
3	28.16	-	29.37	28.56	30.30
4	15.69	-	17.85	15.35	2.05
5	12.33	30.24	26.96	25.43	35.56
6	8.18	22.41	19.18	18.65	26.27
7	11.81	33.24	30.38	30.64	34.18
8	8.97	25.36	23.98	24.68	30.31

## Data Availability

All data are included in the paper.
